# Predictors of Acceptance of Cosmetic Surgery: Instagram Images-Based Activities, Appearance Comparison and Body Dissatisfaction Among Women

**DOI:** 10.1007/s00266-021-02546-3

**Published:** 2021-09-03

**Authors:** Cristian Di Gesto, Amanda Nerini, Giulia Rosa Policardo, Camilla Matera

**Affiliations:** 1 Department of Health Sciences, Section of Psychology, University of Florence, Via San Salvi 12, Pad. 26, Florence, Italy; 2 Department of Education, Languages, Intercultures, Literatures and Psychology, University of Florence, Via di San Salvi, 12–Pad. 26, 50135 Florence, Italy

**Keywords:** Psychological factors, Sociocultural influences, Instagram, Appearance comparison, Body dissatisfaction, Acceptance of cosmetic surgery

## Abstract

**Background:**

This study aimed to test a model in which Instagram images-based activities related to self, friends, and celebrities were associated with acceptance of cosmetic surgery via Instagram appearance comparison and body dissatisfaction. We predicted that Instagram use for images-related activities involving celebrities and self (but not friends) was associated with acceptance of cosmetic surgery both directly and indirectly.

**Methods:**

The study participants were 305 Italian women (mean age, 23 years). They completed a questionnaire containing the Instagram Image Activity Scale, the Instagram Appearance Comparison Scale, the Body Shape Questionnaire-14, the Acceptance of Cosmetic Surgery Scale. A path analysis was performed in which the Instagram images-based activities were posited as predictors of the Instagram appearance comparison, body dissatisfaction and acceptance of cosmetic surgery, respectively.

**Results:**

We found that only image-based activities related to celebrities and self were significantly related to acceptance of cosmetic surgery, whereas friends’ Instagram-related activities were not significantly related to this criterion variable. Moreover, the indirect effect of both Instagram self- and celebrities-images activities on acceptance of cosmetic surgery through Instagram appearance comparison and body dissatisfaction was significant. Friends’ Instagram images-related activities were not associated with acceptance of cosmetic surgery.

**Conclusions:**

Overall, these findings provide information about the role that activities carried out on Instagram, appearance comparison and body dissatisfaction, play on the acceptance of surgery for aesthetic reasons among women. The study highlighted the importance for surgeons to consider some psychological aspects and the influence of sociocultural factors on the interest for cosmetic surgery.

**Level of Evidence IV:**

This journal requires that authors assign a level of evidence to each article. For a full description of these Evidence-Based Medicine ratings, please refer to the Table of Contents or the online Instructions to Authors www.springer.com/00266.

## Introduction

The impact of sociocultural factors on body image ideals has been increasingly recognized in recent years [1]. The Tripartite Influence Model (TIM) of body dissatisfaction emphasizes the role of the media in the onset, development, and maintenance of negative body image [2, 3], fostering interest in body modification strategies such as cosmetic surgery [4]. Several recent studies have shown that Social Network Sites (SNSs) have a significant influence on young women’s body image [5–8]. SNSs have become the most commonly accessed websites on the Internet [9] and their use, as the main channel for social interaction [10], is constantly growing among young adults. Active users on social networks are over 3 billion and 35 million are Italians [11]. Unlike traditional forms of media, such as magazines or movies, SNSs allow users to be not just passive receivers of contents, but also active creators of individual private or public profiles, sharing various forms of personal content, interacting with followers, and viewing, commenting, and “liking” peer-generated content [12–18]. SNSs allow users to receive a constant flow of information (e.g., picture and video sharing, tagging, and newsfeed) from friends as well as celebrities they follow, at any given time as long as they have Internet access on a smartphone, laptops, and desktop computers [19]. Therefore, compared to the traditional media environment, where exposure to content is limited by physical access to material or screen time (e.g., having access to fashion magazines or watching television), the SNSs environment offers young women greater exposure to idealized body in the form of friends’ edited profile picture or celebrities’ latest photos [19].

Through this study we aimed to examine the relationship between the use of Instagram and acceptance of cosmetic surgery via the serial mediation of appearance social comparison and body dissatisfaction.

### Visual Social Media

Instagram is a social networking site used solely for photo and video sharing, which has risen in popularity in recent years, with over 400 million active users [20, 21]. The number of photos uploaded each day on Instagram has increased from 80 million photos per day in 2015 to 95 million photos per day in 2019 [20]. Since Instagram allows individuals to carefully select the personal photos they wish to post and to enhance them with filtering and editing tools [16], a growing body of research has investigated the impact of Instagram use on body image. The use of Instagram for activities such as viewing images focused on body ideals and participating in conversations relating to physical appearance can be particularly harmful for body image. Indeed, engaging in appearance-based activities is associated with concerns about body image [22] and greater self-objectification among young women [5, 12, 23, 24].

Although several studies have reported that the time spent on SNSs and their general use is significantly related to body image concerns [25–27], the type of activity carried out online and the target to which they are addressed (e.g., themselves, celebrities, friends) worth further consideration [22, 26–28]. For example, one study [29] found that it was not the frequency or quantity of Facebook use among adolescents that predicted their levels of body dissatisfaction, but rather the extent to which they engaged in appearance-related activities such as viewing, posting, or commenting on images of themselves or their friends.

### Acceptance of Cosmetic Surgery

Sociocultural theories on body image contend that messaging through media has had a profound effect on how individuals view standards of beauty [30]. Overall, photo-based social media and apps are providing a new reality of beauty for today’s society. These apps allow one to alter his or her appearance in an instant and conform to an unrealistic and often unattainable standard of beauty [31]. SNSs make it easy for individuals to present their “best” selves albeit digitally enhanced [32, 33]. It can be argued that photo-bases SNS, like Instagram, are making us lose touch with reality because we expect to look perfectly primped and filtered in real life as well [34]. The use of editing software can alter an individual’s perception of one’s appearance [35]. Choosing to alter one’s appearance means recognizing a personal perceived imperfection, and this repeated behavior may drive to seek cosmetic care [36, 37]. Indeed, recent studies have shown that the use of social media is associated with increased acceptance of cosmetic surgery [38]. One study [39] experimentally examined whether exposure to images depicting facial cosmetic enhancements increases the desire for cosmetic surgery among young American women. They found that viewing images on Instagram of someone who had undergone cosmetic enhancements directly affected young women’s desire for cosmetic surgery.

Several studies have suggested that body dissatisfaction is one of the factors involved in the decision to surgically modify one's body [4, 39, 40]. Some studies have shown a positive association between body dissatisfaction and acceptance of cosmetic surgery among women, suggesting that people may consider cosmetic surgery as a means to obtain both intrapsychic benefits (e.g., higher self-esteem) and social rewards deriving from appearing more attractive to others [4, 41–43].

### Social Comparison and Body Dissatisfaction

According to the TIM [2, 3], the media influence body dissatisfaction levels through the mediating process of social comparison, that is the tendency to evaluate dimensions of the self, such as body, through comparison with others. Social comparison is also implicated in the attitude toward cosmetic surgery [2, 4, 44, 45]. Women who internalize beauty ideals may be more likely to engage in physical appearance comparison to establish if they meet shared cultural standards of beauty [46, 47]; if physical appearance comparison discloses that a woman does not match the size and shape of other people, that woman may experience body dissatisfaction. Nerini et al. [4] showed that higher levels of physical appearance comparison were positively associated with greater acceptance of cosmetic surgery among young Italian women.

SNSs offer up an ideal platform for social comparison to take place [19, 48]. Indeed, it appears that people are quite interested in learning about others on SNSs, as most networking activity consists of browsing others' profiles without initiating social interaction [49]. Young women explicitly declared that they use SNSs for the purpose of making social comparisons, specifically while viewing others' posts and photos [50]. According to the Social Comparison Theory [51], apart from similar others, people tend to compare themselves with those who are perceived to be better, such as celebrities, who are perceived to be standard bearers of beauty [19, 52]. In their systematic review on the impact of SNSs on body image and disordered eating outcomes, Holland and Tiggemann [16] suggested that engaging in social comparisons mediated the relationship between time online and body image appraisals. Furthermore, in their sample of female university students, Fardouly and Vartanian [26] identified the value of examining physical appearance comparisons where one’s own appearance was perceived to be less appealing or attractive than the one of others. This practice has been associated with higher body dissatisfaction rates [53] and disordered eating [54]. It has been reported that young women engage in this behavior more frequently than young men [55]. Posting personal photos on SNSs seems to make it easier for women to compare their appearance with that of others [22, 24] as individuals tend to share photos of themselves in which they are aesthetically attractive and without any imperfections [56]. Also with regard to Instagram, correlational and experimental studies have shown that appearance comparison with other users mediated the effect of exposure to images of idealized bodies on body dissatisfaction levels [6, 25, 57, 58]. The impact of social comparisons on self-evaluations can vary depending on the comparative target in relation to the self [48, 59].

### The Present Study

The present study aimed to test a statistical model in which Instagram images-based activities related to self, friends, and celebrities were associated with acceptance of cosmetic surgery *via* Instagram appearance comparison and body dissatisfaction. Previous correlational research has highlighted an association between Instagram use and acceptance of cosmetic surgery [37, 38]. However, none of these studies have measured the images-based activities that users can carry out on it in relation to different targets (e.g., themselves, friends, or celebrities). According to Scully and colleagues [48], measuring total time spent on SNSs, which does not account for how this time is spent, is less informative than specifically measuring the use of Instagram for appearance-related activities while logged on it. Moreover, to the best of our knowledge, there are no studies evaluating the relationship between the use of Instagram for images-based activities related to different targets and acceptance of cosmetic surgery.

We predicted that the Instagram image activities related to different targets (self, friends, and celebrities) would not be associated analogously with acceptance of cosmetic surgery. Previous research findings showed that the aesthetic models proposed by celebrities are more desirable than those proposed by friends [48]. Consequently, the Instagram celebrities’ images-related activities (but not the ones related to friends) were hypothesized to be linked to acceptance of cosmetic surgery. The more frequently women expose themselves and interact with perfect, digitally modified appearance-based contents related to celebrities on Instagram, the more favorably they might consider modifying their bodies through cosmetic procedures (Hypothesis 1).

We also hypothesized a link between Instagram images-related activities related to the self and acceptance of cosmetic surgery (Hypothesis 2). The tendency to present the perfect image of self, which can be explained as self-presentation on social media, might include selecting your best photos [13, 60, 61] and greater acceptance of cosmetic surgery to make the virtual self-image real [62].

Furthermore, Instagram appearance comparison and body dissatisfaction were hypothesized to mediate the relationship between Instagram use for images-related activities involving celebrities and self (but not friends) and acceptance of cosmetic surgery (Hypothesis 3). Indeed, people tend to compare themselves with celebrities, who are perceived to be standards of beauty [19]. Much evidence has shown that appearance comparison on Instagram mediated the relationship between exposure to images of idealized bodies and dissatisfaction with one’s body [58]. Several studies have also reported that selfie investment and photo manipulation are significantly associated both with social comparison and greater body dissatisfaction in young adult women [24, 33, 50, 63, 64].

## Method

### Participants

The participants included 305 Caucasian Italian university women aged 19–32 years (M = 23, SD = 2.92). The mean Body Mass Index (BMI) of the sample was 21.78 (*SD* = 3.04), ranging between 15.2 and 33.4. Most of the participants (86.8%) lived in central Italy, 12% in southern Italy or on islands, and 1.2% in northern Italy. Most of them (94.6%) reported being unmarried, whereas 5.4% reported being married or cohabiting. With regard to education, 92.6% of them had high school diplomas, 6.9% had bachelor’s degrees, and 0.5% had master’s degrees. Most of the participants (97.2%) defined themselves as students, whereas 2.8% as workers (1.6% were occasional employees, 0.8% part-time employees, 0.3% were looking for a first job, and 0.1% full-time employees).

### Measures

#### Instagram Use

Participants were asked how much time they spend on Instagram per day (1 = 0–30 min; 12 = 10–11 h). They also rated how often they followed certain types of accounts (health and fitness; celebrities; travels) on Instagram (1 = never; 5 = very often).

#### Instagram Image Activity Scale

The Instagram Image Activity Scale [6] was used to assess the frequency with which various types of imaged-related activities are carried out on Instagram (e.g., posting or watching photos, videos, stories, direct; “liking” photos and videos). This scale consists of 13 items with a three-factor structure: the Activities: self-images subscale measures the frequency with which user can carry out image-based activities related to the self (four items; e.g., “Watching stories or direct in you are there, published by yourself e”; *α* = .85), the Activities: images of friends subscale measures activities related to friends (six items; e.g., “Watching photos or videos where your friends are”; *α* = .77), and the Activities: images of celebrities subscale measures the frequency that a participant carries out image-based activities related to celebrities on Instagram (three items; e.g., “Watching stories or direct where your friends are”; *α* = .86). The scale ranges from 1 (almost never) to 5 (almost always). High scores indicated high use of Instagram for images-related activities.

#### Instagram Appearance Comparison Scale

We adopted the Instagram Appearance Comparison Scale [6] to assess the level to which people make appearance comparisons on Instagram. This fifteen-item scale (e.g., “When I use Instagram, I compare my physical appearance to that of others”) ranges from 1 (never) to 5 (very often). Higher scores represented greater levels of physical appearance comparisons on Instagram (*α* = .94).

#### Body Dissatisfaction

We used the Italian version [65] of the Body Shape Questionnaire-14 [66] to assess female body dissatisfaction. The scale has 14 items (e.g., “I felt ashamed of my body”) rated along a six-point Likert scale (1 = never; 6 = always). The questionnaire asks the participants to respond based on the past 2 weeks prior to administration. High scores indicated greater levels of general body dissatisfaction (*α* = 0.95).

#### Acceptance of Cosmetic Surgery

Acceptance of cosmetic surgery was assessed through the Italian version [67] of the Acceptance of Cosmetic Surgery Scale [68]. This scale is composed of 15 items (e.g., “Cosmetic surgery can be a big benefit to people’s self-image”) rated along a seven-point Liker scale (1 = definitively disagree; 7 = definitively agree). High scores indicated high levels of acceptance of cosmetic surgery (*α* = .93).

#### Sociodemographic Details, BMI, and Previous Cosmetic Surgery Interventions

Each participant reported her age, sex, nationality, educational level, occupational status, and relationship status. We calculated BMIs (kg/m^2^) using the participants’ reported weights and heights. Finally, the participants were asked if they had undergone cosmetic surgery interventions.

### Procedure

Using opportunistic sampling techniques, we recruited the study participants from the School of Psychology at the university with which the authors were affiliated. During regular undergraduate and graduate classes, we asked the students to take part in a study on body image. Participation in the study was voluntary, and we did not provide incentives to the participants. To be eligible for the study, the participants were needed to be 18 years or older women with an active Instagram account. We obtained informed consent from each participant prior to administering the questionnaire. Participants completed measures in paper and pencil format. The questionnaire was anonymous, did not ask for any personally identifiable information, and took about 20 min to complete. The Ethical Committee of the University of Florence approved the study procedures. All procedures performed in studies involving human participants were in accordance with the ethical standards of the institutional and/or national research committee and with the 1964 Helsinki Declaration and its later amendments or comparable ethical standards.

### Data Analyses

First, descriptive statistics and intercorrelations between all the variables were calculated. Second, we examined the fit of a statistical model in which the Instagram images-based activities related to self, friends, and celebrities were posited as predictors of the Instagram appearance comparison, body dissatisfaction and acceptance of cosmetic surgery, respectively. BMI was included to control for its effect, and the Instagram self, friends, and celebrities’ images activities were allowed to covary. Less than 1% of the data were missing. We used a mean imputation process to replace the missing values. All the assumptions for path analysis were satisfied [69]. The hypotheses were tested using Amos (version 22); we used bootstrapping to test mediation by estimating the presence and size of the indirect (i.e., mediated) effects [70]. The sample size in the present study was bigger than the recommended size of 200 participants [71]. We adopted the maximum likelihood procedure to derive the parameter estimates and used the following goodness-of-fit indices: the χ2/df ratio, a good score of which is 2 or below; the comparative fit index (CFI); the Tucker-Lewis Index (TLI); the Incremental Fit Index (IFI), the value of which should be higher than 0.95; the Normed Fit Index (NFI), a good score of which is more than 0.90; the Root Mean Square Error of Approximation (RMSEA); a 90% confidence interval for RMSEA (RMSEA 90% CI); and the standardized root mean square residual (SRMR). RMSEA and SRMR are considered acceptable if they are 0.08 or lower [72].

## Results

All participants (100%) reported having their own active Instagram account. Most of them (36.7%) used Instagram for 1–2 h, 23.6% for 30 min–1 h, 14.1% for less than 30 min, 11.8% for 2–3 h, 5% for 3–4 h, 4.9% for 5-6 h and only 3.9% for 10–11 h per day. Moreover, most of the participants (38.8%) reported that they often followed health and fitness-related accounts, celebrities-relates accounts (36.9%) and travels-related account (24.3%). None of the participants reported having undergone previous cosmetic surgery.

Table 1 shows the descriptive statistics (means and standard deviations) and the intercorrelations among Instagram images-based activities (self-images, images of friends, and images of celebrities), appearance comparison on Instagram, body dissatisfaction, and acceptance of cosmetic surgery. The data were normally distributed (skewness <0.90; kurtosis <2.75), as the skews for all variables were lower than 2 and kurtosis is lower than 7 [73].

**Table 1:** Mean (*M*), Standard Deviation (*SD*) and intercorrelations between all variables

*Variable*	1	2	3	4	5	6	7	*M* (*SD*)
1. BMI	1							21.78 (3.04)
2. Activities: self-images	.02	1						2.98 (1.04)
3. Activities: images of friends	.01	.53***	1					3.45 (.75)
4. Activities: images of celebrities	.11	.36***	.23***	1				3.21 (1.16)
5. Instagram Appearance Comparison	.22***	.43***	.21*	.28***	1			2.88 (.92)
6. Body Dissatisfaction	.47***	.29***	.16**	.22**	.69***	1		3.11 (1.35)
7. Acceptance of Cosmetic Surgery	.10	.39***	.18*	.31***	.35***	.38***	1	3.05 (1.30)

N = 305; * p < .05 **p < .01 ***p < .001

On average, the participants carried out more activities on Instagram based on images of friends, followed by images of celebrities and, finally, by self-images. They reported average levels of both appearance comparison on Instagram and body dissatisfaction, and low levels of acceptance of cosmetic surgery. From the correlation analyses (table 1), we can observe that the use of Instagram for image-based activities, regardless of the specific type of activity performed on it, was positively associated with higher levels of Instagram appearance comparison, body dissatisfaction and acceptance of cosmetic surgery.

**Figure 1:**
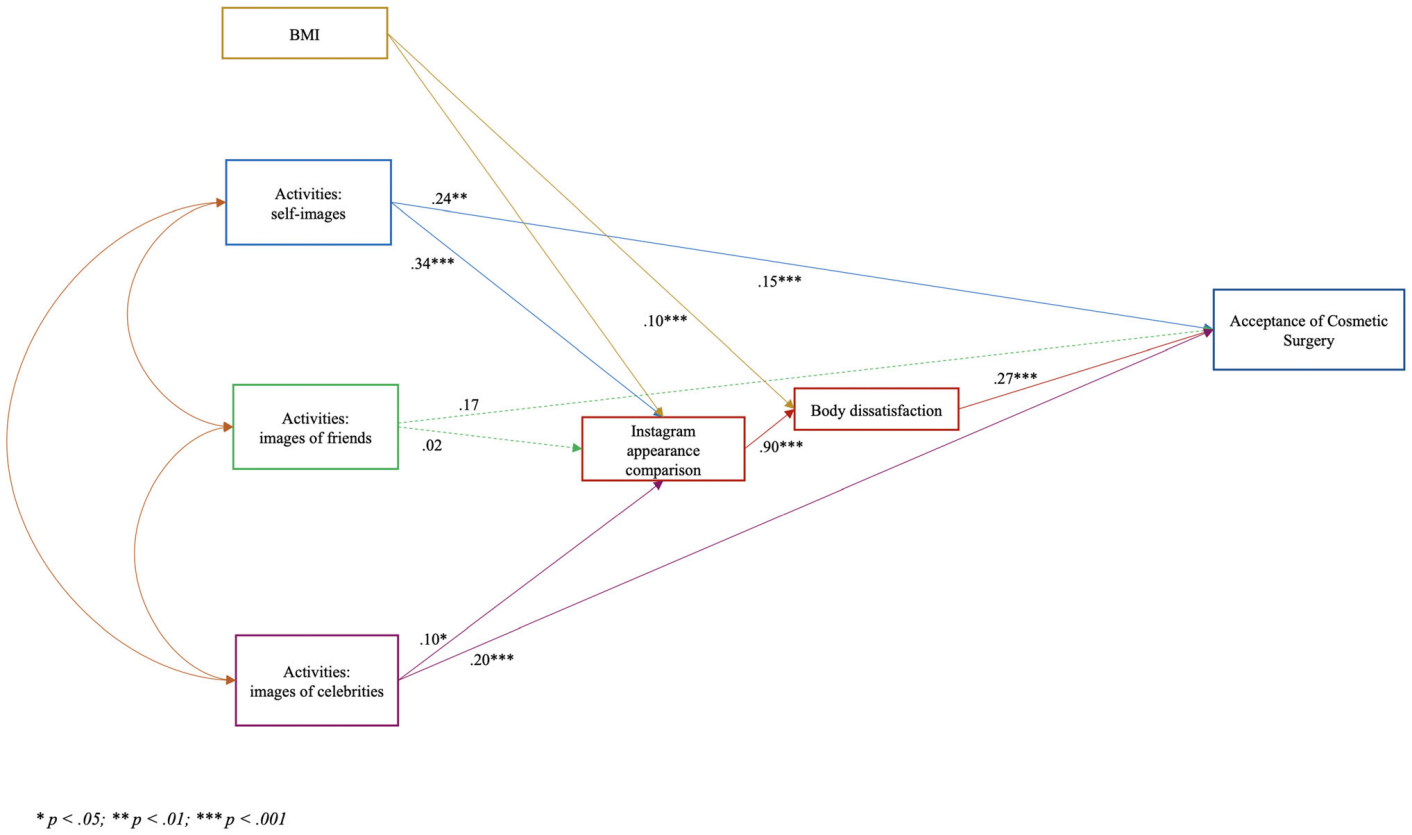
Mediation model

The statistical model (Figure 1) fitted very well with the data [*χ2* = 8.53, *p* = .38; *χ2*/df = 1.06; RMSEA = .01 (CI = .00; .07); SRMR = .02; CFI = .99; TLI = .99; IFI = .99; NFI = .99]. Covariances ranged between .20 (*p* < .001) and .43 (*p* < .001).

Hypotheses 1 and 2 were confirmed. Only image-based activities related to self and celebrities were significantly (and positively) related to acceptance of cosmetic surgery, whereas friends’ Instagram-related activities were not significantly related to this criterion variable.

In line with Hypothesis 3, the bootstrapping procedure [74] showed that the indirect effect of both Instagram self- and celebrities-images activities on acceptance of cosmetic surgery through Instagram appearance comparison and body dissatisfaction was significant (self-image activities: .067; 95% CI: .039; celebrities-image activities: .019; 95% CI: .001; .042). Friends’ Instagram images-related activities were not associated with acceptance of cosmetic surgery either directly or indirectly.

The statistical model accounted for much of the variance in body dissatisfaction (57%) and for a satisfactory percentage of the variance of acceptance of cosmetic surgery (26%).

## Discussion

The present study examined the relationship between specific Instagram image-related activities and acceptance of cosmetic surgery among young Italian women as well as its underlying mechanisms. Our findings showed that young women’ acceptance of cosmetic surgery was significantly associated with some of the Instagram image-related activities. Consistently with our predictions, Instagram celebrities and self-image related activities were directly associated with acceptance of cosmetic surgery.

Regarding to Instagram celebrities’ images, it seemed that the exposure to and the interaction with the enhanced photos, videos, or stories of celebrities on Instagram that promote beauty standards that are unattainable in a natural way may trigger assumptions that these photos are indicative of how they actually appear. As these ideals are highly desired by women, more than those proposed by other sociocultural sources (e.g., friends) [48], women are likely to seek ways to achieve these standards. Cosmetic surgery might be conceived as a socially acceptable way to achieve such ideals of aesthetic beauty due to the promotion of surgery as a method of intervening on the body in a quick and accessible way [37]. Indeed, several social media celebrities are actively advertising surgery as a strategy their followers can use to achieve the ideal body [75]. Moreover, many people nowadays choose cosmetic surgery as a life changing gift [76] and many patients carry images of celebrities to their consultations to emulate their attractive features [34]. Finally, Instagram friends’ images-related activities were not significantly related to acceptance of cosmetic surgery directly (Hypothesis 1). In line with Scully et al. [48] the beauty ideals proposed by celebrities are more desirable than those proposed by friends which are instead more realistic and obtainable.

With regard self-image-related activities, the steps before posting personal photos, videos, or stories (e.g., picture taking, selection, and editing) may be a modern form of body checking through which changes in weight and appearance can be identified and tracked [33, 77, 78]. During these activities young women can carefully check their body image, comparing it with sociocultural standards and thinking about how to modify it to be closer to their beauty ideal [79, 80]. Consistently with some previous studies, our findings suggest that the tendency to present a perfect self-image made it more likely to consider cosmetic surgery as a means of appearing more attractive [4, 38, 62]. These findings are in line with a recent study showing that the primary reason for women patients seeking cosmetic surgery is the desire to look better in photographs and videos that portray them [36]. This is an alarming trend because filtered selfies often present an unattainable look and are blurring the line of reality and fantasy for these patients [34].

Moreover, in line with Hypothesis 3 about the mediational role of Instagram appearance comparison and body dissatisfaction, our findings showed the activities conducted on Instagram related to images of celebrities were associated to the acceptance of cosmetic surgery not only directly but also indirectly. Celebrities represent an unattainable, psychologically distant, and extreme target of comparison for adolescent girls and young women [48]. Using Instagram for celebrities-related activities was positively associated with the tendency to evaluate one’s body in comparison with the idealized and digital enhanced physical appearance of celebrities that, in turn, was associated with women’s acceptance of cosmetic surgery. Our findings are consistent with studies that showed how people more inclined to judge their own aesthetic appearance in relation to the unrealistic physical attractiveness of others may experience feelings of body-related distress, such as body dissatisfaction [81, 82], making them more interested in cosmetic surgery to enhance their physical appearance [14].

Furthermore, we found that also self-appearance activities were associated with acceptance of cosmetic surgery both directly and indirectly relationship. We can observe that Instagram activities related to self were linked with the tendency to compare one's physical appearance with that of others, which could help establish if one is effectively meeting sociocultural standards of beauty [46, 47]. If physical appearance comparison discloses that a woman does not match the beauty ideal which she wants to achieve, that woman may experience body dissatisfaction [83–85]. Concerns about physical look could make people more interested in cosmetic surgery to enhance their appearance [62].

Finally, regarding to Instagram activities about friends, our results do not reveal a direct or indirect link between these activities and acceptance of cosmetic surgery. It seemed that the greater realism of the aesthetic standards proposed by friends, compared to those of celebrities, on Instagram would neither favor physical appearance comparison and body dissatisfaction nor a greater acceptance of cosmetic surgery. Indeed, the impact of social comparisons on self-evaluations can vary depending on the comparative target’s distance, extremity, and attainability in relation to the self [59].

The statistical model we tested explained a good percentage of variance in body dissatisfaction levels (57%) and a satisfactory percentage of variance (26%) in the acceptance of cosmetic surgery. From a conceptual point of view, it should be considered that we have analyzed a specific sociocultural factor (i.e., Instagram use) as a possible predictor of the acceptance of cosmetic surgery among young women; however, the sociocultural factors that can play a role in this acceptance are manifold (e.g., traditional media, peer, family, partner). Furthermore, there are other factors, in addition to sociocultural ones, that can favor both dissatisfaction with one’s body and the acceptance of cosmetic surgery. Among these, individual variables could be relevant (e.g., self-monitoring, self-awareness, control beliefs over one's own physical appearance). For these reasons, it seems reasonable to us to argue that the variance explained by Instagram images-based activities appears to be good for body dissatisfaction and satisfactory for interest in cosmetic surgery. This conceptual reflection can also be supported from a statistical point of view, considering that variance explained from .05 to .10 indicates a very small score, a score of > .20 is medium and relatively satisfactory, a score of > .30 indicates a large value of variance explained, a score of > .40 or greater is very large [86].

This study has some limitations. First, because of the correlational nature of this research, we cannot make causal inferences. Future research in SNSs, body image and cosmetic surgery could adopt experimental designs to investigate whether different activities conducted on Instagram are causally linked with levels of acceptance of cosmetic surgery. Second, we assessed acceptance, but not effective engagement, of cosmetic surgery. Perspective and experimental studies must clarify the causal relationship between the variables and should examine the relationship between attitudes and the actual decision to undergo cosmetic surgical procedures. Moreover, we used a convenience sample, so that our findings are not generalizable to the entire population. Previous research has also found that the relationships between variables outlined in sociocultural models of body dissatisfaction vary depending upon the age, gender, and sociocultural setting of the participants. For example, cultural specificities are important factors to consider when devising and analyzing sociocultural models of body dissatisfaction, with the focus on appearance potentially varying between countries [44]. Further attention is warranted to extrapolate how the effects of online media exposure can vary depending on an individual’s sociocultural environment, gender, and age.

Finally, this study is not exhaustive of potential variables that may increase interest for cosmetic surgery by women. Future studies could examine if body compassion, which is negatively related to body dissatisfaction and positively with acceptance of the perception of personal inadequacies, failures, and difficulties related to the body [87], might be a relevant mediator of the relationship between the Instagram use and acceptance of cosmetic surgery. Considering the proliferation of research within the positive body image literature, future research could also benefit from examining protective factors that may buffer young girls from the more adverse effects of social media sites. Emerging research has highlighted how young women who were exposed to body-positive posts experienced improvements in mood, body satisfaction, and body appreciation in comparison to being exposed to thin ideal and appearance-neutral posts [88]. Future research could also examine the transactional and reciprocal effects of social media. It is uncertain whether vulnerability factors such as high levels of appearance comparison or body dissatisfaction predispose individuals to seek out content that favor the consideration of cosmetic surgery, or whether SNSs such as Instagram cause individuals to engage in such consideration due to the features they afford which may heighten their levels of social comparison and body dissatisfaction.

## Conclusions

The present study suggests that frequent use of Instagram for activities related to the self and celebrities may favor acceptance of cosmetic surgery fostering physical appearance comparison and body dissatisfaction. These sociocultural and psychological processes are all important in explaining how young women’ online appearance-related activities are related to favorable attitudes toward strategy aimed to modify permanently one’s physical attributes.

From a practical point of view, these findings could help plastic surgeons in their practice, suggesting the importance of psychological assessment to investigate if women’s motivation to cosmetic procedures is related to stable reasons or is determined by temporary elements [4, 89, 90], such as Instagram’ physical appearance ideals. Candidates for surgery for purely cosmetic reasons, focusing on the potential short-term benefits of such interventions, may not be fully aware of the risks such interventions can have on a psychophysical level. Surgeons could carry out a preliminary assessment aimed at investigating patients' expectations about the outcome of the surgery, proposing more realistic one; they might explore if they feel compelled to achieve sociocultural aesthetic ideals, emphasizing their unrealistic and unattainable nature. Moreover, they might be reminded that cosmetic surgery does not necessarily help women to improve their body image evaluation [91, 92]. If women modify their bodies through cosmetic surgery without changing attitude toward their body self, concerns about their appearance may not decrease, which could lead them to look for further cosmetic procedures, without ever feeling comfortable with their own body image.
